# Supplementary material for: MetaRibo-Seq measures translation in microbiomes
Source: Nat Commun. 2020 Jun 29;11:3268. doi: 10.1038/s41467-020-17081-z (PMC7324362; doi:10.1038/s41467-020-17081-z)
Supplement: Supplementary file 10 — Supplementary Data 7 [file 41467_2020_17081_MOESM10_ESM.zip › File2/Confidence_High_Taxonomy/254113_out.krona.html]

Javascript must be enabled to view this page.

members
magnitude
magnitudeUnassigned
count
unassigned
taxon
rank

254113\_out

2

2
superkingdom
2

phylum
2
1239

2
class
186801

186802
2
order

1
family
541000

1
1263
genus

1

SRS143722\_contig\_number\_contig-100\_20811.54248
species
46228

1
543314
family

86331
1
genus

1

SRS104165\_contig\_number\_12099
species
114527
